# The Current Role of Hydroxyurea in the Treatment of Sickle Cell Anemia

**DOI:** 10.3390/jcm13216404

**Published:** 2024-10-25

**Authors:** Montserrat López Rubio, María Argüello Marina

**Affiliations:** 1 Servicio de Hematología y Hemoterapia, Hospital Universitario Príncipe de Asturias, 28805 Madrid, Spain; mlrubio@salud.madrid.org; 2 Servicio de Hematología y Hemoterapia, Hospital Universitario de Guadalajara, 19002 Guadalajara, Spain

**Keywords:** sickle cell disease, sickle cell anemia, hydroxyurea, hydroxycarbamide, pregnancy, breastfeeding, oncogenic effects

## Abstract

Despite advancements in treatment of sickle cell disease (SCD), hydroxyurea, a ribonucleotide reductase inhibitor, remains the cornerstone of therapy. While its primary effect is the elevation of fetal hemoglobin (HbF), hydroxyurea’s mechanisms of action are multifaceted. Hydroxyurea (HU) reduces leukocyte and platelet counts, decreases the expression of endothelial adhesion molecules CD36 and CD49d, and increases nitric oxide and cyclic nucleotide levels, which may facilitate vascular dilation and further HbF induction. Numerous studies have demonstrated that hydroxyurea therapy reduces the frequency of painful episodes, acute chest syndrome, and the need for erythrocyte transfusions and hospitalizations. Long-term use of hydroxyurea leads to reduced morbidity and mortality. Hydroxyurea should be initiated in children from 9 months of age, including asymptomatic individuals, and is recommended for adults experiencing pain crises that significantly interfere with daily activities or quality of life, as well as those with severe or recurrent vaso-occlusive crises, ACS, or severe symptomatic anemia. Hydroxyurea is not recommended during pregnancy or lactation due to potential teratogenic effects and transfer into breast milk. However, its use may be considered in high-risk patients, particularly during the second and third trimesters. Concerns about secondary tumor development have not been substantiated in long-term follow-up studies. Alternative therapies, including L-glutamine, crizanlizumab, and voxelotor, are not presently approved or available for clinical use in Europe.

## 1. Introduction

Sickle cell disease (SCD) is an inherited disease caused by a mutation in the beta-globin gene that leads to the formation of HbS instead of HbA, which in sufficient proportions and under conditions of deoxygenation polymerizes and leads to the deformation of red blood cells, which affects their flexibility and adherence to the vascular endothelium [1,2], causing both acute and chronic complications. Over 7 million people are affected by SCD worldwide, and it is a leading cause of childhood mortality, especially in children under five years of age. It also reduces life expectancy in affected adults [3].

Comprehensive management of SCD is crucial and includes dietary hygiene measures, vaccination, genetic counseling, specialized monitoring based on organ involvement, and management of acute and chronic complications [4]. Despite advances in treatment, hydroxyurea (HU) remains the primary therapy since its pivotal trial in 1995, which showed a reduction in vaso-occlusive crises (VOCs) and blood transfusions [5]. Subsequent studies have reported decreased mortality in patients treated with HU [6,7].

In light of recent advances in understanding the pathophysiology of SCD and the approval of new therapeutic agents, it is essential to re-evaluate the role of HU in contemporary disease management. This review aims to present up-to-date clinical evidence on the efficacy and safety of HU. To accomplish this, a comprehensive literature search was performed using databases from the Cochrane Library and PubMed, covering publications through 2024.

## 2. Treatment of Sickle Cell Disease and the Role of Hydroxyurea

### 2.1. Mechanism of Action

HU is a cytostatic agent that inhibits ribonucleotide reductase, causing a temporary halt in hematopoiesis, followed by a phase of “stress erythropoiesis”. This phase recruits early erythroid progenitors capable of producing fetal hemoglobin (HbF) [8,9]. As a result, there is an increase in HbF production and a decrease in the percentage of sickle hemoglobin (HbS) in red blood cells, which inhibits polymerization and prevents sickling. The exact mechanisms by which HU induces HbF, the principal pharmacodynamic effect, remain not fully understood [4,10]. The effects of HU may, in part, be mediated through epigenetic modifications, specifically DNA methylation. However, the current body of research on these modifications and their clinical implications remains limited and requires further investigation.

HU also reduces the expression of endothelial adhesion molecules like CD36 and CD49d, decreases white blood cell and platelet counts, and increases nitric oxide and cyclic nucleotide levels, which may facilitate vascular dilation and induce HbF. These effects reduce episodes of pain, acute chest syndrome (ACS), hospital admissions, and the need for transfusions in SCD patients [9,11,12,13,14].

### 2.2. Clinical Benefit

HU has been extensively studied in clinical trials for its potential to reduce complications in patients with SCD. Four notable trials have compared HU to placebo in different patient populations, providing valuable insights into its efficacy and safety.

The “Multicenter Study of HU (MSH)”, published in 1995 (NCT00305175, Enrollment Status: Completed), was a double-blind, randomized trial conducted in the United States. This study enrolled 299 adults with SCD and evaluated the effects of HU over a two-year period, with doses adjusted to the maximum tolerated level. The primary objective was to assess whether HU could reduce the incidence of VOCs, ACS, and hospitalizations in adult patients. The results demonstrated a significant reduction in annual VOC rates, with a mean decrease of −2.80 crises per year (*p* = 0.005) and −1.50 crises requiring hospitalization (*p* = 0.007). Moreover, the time to the first crisis was significantly longer in the HU group. However, the study’s limitations include its focus on adult patients, limiting its applicability to pediatric populations, and its relatively short follow-up, which may not have fully captured the long-term safety and efficacy of HU [5].

The “Belgian Study”, published in 2005, was a randomized, placebo-controlled trial involving 25 children and young adults with HbSS. This trial aimed to evaluate the long-term efficacy of HU in reducing painful crises, hospitalizations, and the need for transfusions over a 10-year period. HU was initiated at a dose of 20 mg/kg/day, increased to 25 mg/kg/day if tolerated, with a crossover design after six months. The study found that HU significantly reduced hospitalizations for painful episodes in 73% of patients, compared to 14% in the placebo group (*p* < 0.001). Despite these promising results, the study was limited by its small sample size and single-center design, restricting the generalizability of the findings to larger or more diverse populations. Furthermore, long-term follow-up data were limited, preventing comprehensive evaluation of the effects of prolonged HU use [15]. Patients with very severe disease could also reduce the applicability of the findings to higher-risk populations [16,17].

In 2012, a randomized, double-blind trial in India (enrollment status completed) included 60 children aged 5 to 18 years with SCD and frequent VOCs. The study aimed to assess the efficacy of HU at a low fixed dose (10 mg/kg/day) in reducing VOCs, transfusions, and hospitalizations. The trial lasted 18 months. HU at a low fixed dose of 10 mg/kg/day significantly reduced VOCs by a mean of −9.60 events (95% CI −10.86 to −8.34; *p* < 0.00001). The need for transfusions and hospitalizations was also significantly reduced (*p* < 0.001). However, the relatively short duration of the study may limit the understanding of HU’s long-term effects in this population [18].

A recent meta-analysis, published in 2024, examined the role of HU in preventing organ damage in individuals with SCD. This analysis included 45 articles published from 2003 to 2023. It demonstrated that a daily dosage of 20 mg/kg of HU leads to an 18% increase in HbF, which is associated with significant clinical benefits. These benefits include reductions in transcranial Doppler velocity (*p* < 0.0001), tricuspid regurgitant velocity (*p* = 0.01), albuminuria, and splenic abnormalities [19].

### 2.3. Indications

In 1998, the U.S. Food and Drug Administration (FDA) approved HU for the treatment of SCD in adults. Subsequently, in 2017, the FDA expanded this approval to include pediatric patients aged 2 years and older. HU is currently recommended for any patient with genotype SS or Sβ^0^ thalassemia from 6 to 9 months of age, to be administered indefinitely [4,20]. Early initiation of HU reduces or prevents chronic organ damage, particularly cerebral, renal, and splenic injuries [21]. Several studies have shown that HU treatment preserves splenic and cerebral function and does not cause growth delays or serious toxicities in children.

For example, a pilot study by Wang et al. in 2001 included 28 patients with a mean age of 15 months receiving HU for two years at a fixed dose of 20 mg/kg/day. The study showed a delay in functional asplenia, with only 47% of patients affected, compared to 80% in the literature [22]. Similarly, a single-center retrospective study published in 2009 by Jane S. Hankins et al. involved 43 children. It showed that 14% fully recovered splenic function, and 5% preserved it after a median of 2.6 years on HU at the maximum tolerated dose. Additionally, 96% of children who underwent magnetic resonance angiography before and during treatment showed no changes in cerebral ischemic lesions after a median of 2.9 years of treatment [21]. A sub-analysis of the Baby Hug clinical trial published in 2011 did not achieve statistical significance in the primary endpoint (qualitative splenic uptake on 99mTc scintigraphy), but it suggested benefits for splenic function [16,17].

Recent studies support earlier HU administration, such as the 2019 publication by Schuchard SB et al., which included 65 patients categorized into three age groups. Group 1, with a mean age of 7.2 months, had higher hemoglobin levels, higher mean corpuscular volume, and lower reticulocyte counts at 24 months compared to Group 3, with a mean age of 35.5 months. Group 1 also had higher HbF levels and fewer hospitalizations, VOCs, and transfusions [23]. In 2019, Ware RE et al. highlighted that initiating HU therapy before the first year of life significantly improves clinical and laboratory outcomes. They recommended prescribing HU from 5 to 12 months of age to maximize benefits and prevent severe complications [24].

Concerning brain function and indicators within the central nervous system (CNS), the TWiTCH trial, conducted by Ware et al., was a multicenter, Phase 3, open-label, non-inferiority trial published in 2016. The trial aimed to compare HU with chronic blood transfusions in maintaining transcranial Doppler (TCD) flow velocities in children with SCD. The study included children aged 4 to 16 years who had abnormal TCD flow velocities (≥200 cm/s) but no severe vasculopathy. A total of 121 participants were enrolled and randomly assigned to receive either standard transfusion therapy or HU.

The primary endpoint was the change in TCD velocities after 24 months. The results demonstrated that HU was non-inferior to transfusion therapy, with TCD velocities averaging 138 cm/s in the HU group compared to 143 cm/s in the transfusion group. Notably, no strokes were reported in either treatment arm throughout the trial, highlighting that HU can effectively maintain TCD velocities and serve as a viable alternative to chronic transfusion therapy.

This finding is particularly important, as it suggests a less invasive and potentially more accessible option for stroke prevention in this high-risk population. The use of HU not only presents a credible alternative to reduce the need for blood transfusions in routine clinical practice, it also holds significant promise in resource-limited settings. By decreasing reliance on transfusion protocols, HU offers effective stroke prevention without the associated complications of regular transfusions [25].

A recent study by Casella et al. (2024) has highlighted the potential neuroprotective effects of HU in children with SCD. This prospective study included children aged 12 to 48 months with normal neurological examinations and aimed to determine whether HU could reduce the incidence of CNS injuries by 50%. The study enrolled a total of 12 participants, divided into two groups (HU vs. placebo), with six children in each group. Among the participants, one child in the HU group experienced a primary outcome event (CNS injury), compared to four in the placebo group, suggesting an approximate 80% reduction in the incidence of CNS injuries associated with HU. However, statistical significance was not achieved (*p* = 0.2914). Additionally, the Stroke Consequences Risk Score (SCRS) was lower in the HU group (mean score of 0.078) compared to the placebo group (0.312), although this difference also lacked statistical significance (*p* = 0.072). The absence of statistical significance in both cases was likely due to the small sample size [26].

Similarly, another study, published in 2024 by Lai et al., explored the effects of HU on working memory in children with SCD. This prospective study included children aged 7 to 18 years diagnosed with SCD and assessed their working memory before and one year after initiating HU treatment. Using functional magnetic resonance imaging (fMRI) during n-back tasks, which require participants to recall previously presented stimuli, significant changes were observed in the activation of brain regions related to working memory. These findings suggest that HU has a positive impact on neurocognitive function in children with SCD. Consequently, the results of this study indicate that HU is not only effective in reducing hematological complications but also plays a critical role in preserving cognitive function, particularly working memory [27].

In conclusion, these studies emphasize the importance of considering HU as a comprehensive treatment strategy for children with SCD. The evidence suggests that early initiation of HU therapy could maximize its benefits, not only improving clinical outcomes but also enhancing quality of life and academic performance. This highlights the need for timely intervention to achieve better long-term health outcomes and educational achievements in affected children.

In adults, HU treatment should continue if initiated in childhood. It is recommended for symptomatic homozygous HbSS or HbSβ^0^ thalassemia patients experiencing VOCs, priapism, symptomatic anemia, renal disease, proteinuria, organ damage, pulmonary hypertension, or stroke, in the absence of chronic transfusion therapy [4,20].

There is no consensus on HU treatment for other SCD variants, such as HbSC disease. While HbSC disease is generally less severe than the HbSS and HbS-β^0^ thalassemia genotypes, many HbSC patients experience significant complications. Despite nearly 30 years of demonstrated efficacy of HU in adult SCD patients, the safety and efficacy of HU in HbSC patients remain uncertain. A multicenter, placebo-controlled Phase 3 clinical trial is needed to determine whether HU therapy is effective for individuals with HbSC disease [28].

In the context of SCD in the S/D Punjab genotype, it is the third most common genotype observed in Oman and is linked to recurrent VOCs, acute chest syndrome (ACS), and avascular necrosis. Recent research has demonstrated that treatment with HU does not correlate with improved prognosis or enhanced survival outcomes in individuals with this genotype. This contrasts with the well-documented benefits of HU in other SCD genotypes, where it has been shown to reduce complications and improve the overall quality of life [29].

### 2.4. Dosage

The recommended initial dose is 15–20 mg/kg/day orally in a single dose (if the glomerular filtration rate is below 60 mL/min, the initial dose should be adjusted to 5–10 mg/kg/day).

In children over 5 years of age, the dose should subsequently be increased by 5 mg/kg/day every 2 months until the maximum tolerated dose (MTD) is reached or a maximum dose of 30–35 mg/kg/day is attained [4,9,30].

Although the current guidelines recommend increasing the dose to the MTD, fixed low doses are often used in resource-limited countries. A meta-analysis published in 2024 evaluated the efficacy of escalated versus fixed low doses of HU in adults with SCD. Nine studies were included: four involving fixed low doses, and five with escalated doses. No difference was found in the rate of VOCs between the two groups (*p* = 0.73). The mean difference in hemoglobin from baseline to follow-up was greater in the studies with fixed low doses (1.07 g/dL vs. 0.54 g/dL, *p* = 0.01). Despite the limited number of studies, a balance appears to exist regarding the most appropriate HU dosing regimen in adults with SCD. This study holds particular importance within the context of SCD, which remains highly prevalent in many low-resource countries that lack the infrastructure required for comprehensive patient management. By comparing escalated and fixed low doses of HU, this research offers critical insights that may inform treatment strategies in settings where monitoring capabilities and resources are constrained. Optimizing dosing regimens has the potential to improve patient outcomes and contribute to narrowing the disparity in care between developed and developing regions. To advance this field, controlled studies evaluating HU at maximum tolerated doses versus fixed low doses in adults with SCD are crucially needed [31].

Regarding dosing in children, the HUGKISS study compared two HU dosing strategies in children under 3 years of age with SCD: a fixed dose of 20 mg/kg/day, and an MTD that could increase up to 35 mg/kg/day. This study showed that the MTD achieved greater increases in HbF and total hemoglobin levels, as well as a more significant reduction in white blood cell counts and other disease-related markers. Although the MTD was associated with a higher incidence of neutropenia, no significant differences were observed in the rates of severe infections between the two groups. The study’s findings confirm the feasibility of administering the MTD in very young children, suggesting that future larger trials could validate the clinical benefits of initiating treatment with higher doses of HU at an early age [32].

Recommendations for appropriate treatment with HU are summarized in Table 1.

Oxygen gradient ektacytometry is a valuable technique for quantifying red blood cell (RBC) deformability, offering a comprehensive assessment of multiple RBC characteristics. These include mean corpuscular volume (MCV), mean corpuscular hemoglobin concentration (MCHC), HbF percentage (HbF%), and HbS percentage (HbS%). Together, these parameters significantly influence the sickling behavior of RBCs, particularly in response to HU treatment. The ektacytometry curves, along with their associated parameters—specifically, the point of sickling (PoS) and the minimum elongation index (EI-min)—provide a clear representation of the improvements in RBC properties and the reduction in sickling propensity resulting from HU therapy. This technique proves especially useful in clinical scenarios, such as evaluating non-responders to treatment or assessing the efficacy of combination therapies [33].

Suboptimal medication adherence is a well-documented issue, particularly prevalent among younger patients, which can significantly impact clinical outcomes. Recent research has investigated the various underlying causes contributing to this problem [34] and has implemented strategies such as digital monitoring through mobile phone applications [35] and enhanced oversight by pharmacists [36] to improve treatment adherence. Although the results of these interventions have been mixed, a multidisciplinary approach involving pharmacist support has generally shown promising outcomes. These findings underscore the necessity of individualized strategies to address adherence challenges and optimize patient management. Continued research is essential to further evaluate the effectiveness of current interventions and explore novel approaches to improving adherence, particularly within pediatric populations.

## 3. Safety Profile

HU is a well-tolerated drug with few short- and long-term toxicities. The most common short-term toxicity is cytopenia. Although all cell lines may be affected, the most frequent hematological toxicity is mild-to-moderate neutropenia, followed by reticulocytopenia and thrombocytopenia [4,9].

Myelosuppression is an expected side effect of HU and is used to determine the MTD, which is the optimal maintenance dose for preventing long-term organ damage [4,9,37,38]. Other adverse effects that may occur with HU treatment include oral ulcers, nausea, diarrhea, rash, hyperpigmentation, nail changes, or malleolar ulcers, as well as impairment of fertility, teratogenicity, and mutagenicity [4].

Treatment adjustment criteria based on the occurrence of adverse effects are summarized in Table 2.

## 4. Hydroxyurea and Fertility

In males, HU has been linked to reduced sperm counts, which may persist for over a year after stopping the therapy [39]. In females, there is evidence of diminished ovarian reserve and an increased risk of teratogenicity during pregnancy. HU use in pregnancy is associated with higher risks of miscarriage, stillbirth, and low birth weight [40]. Early exposure to HU in children with SCD may potentially compromise fertility [41].

A recent systematic review and meta-analysis evaluated fertility parameters in both pediatric and adult SCD patients undergoing HU therapy. The analysis included four studies on sperm parameters and four on anti-Müllerian hormone levels and ovarian reserve. The findings indicated compromised fertility, with significant deviations from baseline values. In males, HU therapy was associated with a reduction in sperm concentration, which persisted after treatment cessation. HU also led to sustained reductions in total sperm counts, although sperm volume, motility, and morphology were unaffected. In females, HU therapy was linked to decreased mean AMH levels, with 18.2% of patients showing evidence of reduced ovarian reserve [42].

In a recent study conducted by Diesch-Furlanetto et al., a comprehensive histological analysis of ovarian follicle density was performed on a cohort of 76 girls and young women undergoing ovarian tissue cryopreservation prior to hematopoietic stem-cell transplantation (HSCT). The objective of the study was to assess the impact of HU on ovarian reserve. Of the total participants, 46% had received HU treatment, with a median daily dose of 23 mg/kg and a median treatment duration of 44 months. The results demonstrated no statistically significant differences in the density of growing follicles between the group treated with HU and the untreated group. These findings suggest that HU may not have a detrimental effect on ovarian follicle density in this patient population, offering important insights for fertility preservation strategies in young women undergoing HSCT [43].

## 5. Hydroxyurea in Pregnancy

The pathophysiology of SCD worsens during pregnancy, making it a high-risk period for both the mother and fetus, maternal morbidity and mortality rates being 5 to 10 times higher compared to pregnant individuals without SCD. In low- and middle-income countries, these mortality rates can be at least 20 times higher [39].

Acute chest syndrome (ACS) and pneumonia occur in 6.5% of pregnant women with SCD, with pulmonary complications accounting for 88% of maternal deaths in this population. Severe morbidities associated with SCD during pregnancy include an increased incidence of bacterial infections (odds ratio [OR], 2.48), pulmonary thromboembolism (relative risk [RR], 7.74), and pulmonary hypertension (OR, 6.3). Additionally, obstetric complications are more prevalent in pregnant patients with SCD, including preeclampsia (RR, 2.06), eclampsia (OR, 3.02), cesarean delivery (OR, 1.54), placental abruption (OR, 1.6), and preterm labor (OR, 1.4) [25,26,27]. Perinatal risks are similarly heightened in pregnancies complicated by SCD, with increased occurrences of fetal growth restriction (OR, 2.79), low birth weight (OR, 2.00), small-for-gestational-age neonates (RR, 3.72), and preterm birth (RR, 2.21). Furthermore, there is a higher risk of stillbirth (OR, 4.05) and neonatal death (OR, 2.71) [40].

Clinical practice often involves discontinuing HU therapy before or at conception due to concerns about fetal effects. As a result, most pregnant individuals with SCD manage their condition without disease-modifying treatments, relying on intermittent blood transfusions, which have their own risks.

Safety concerns regarding the use of HU during pregnancy primarily originate from animal studies conducted over 50 years ago. These studies demonstrated unacceptable levels of fetal toxicity at plasma concentrations significantly higher than those administered to humans, particularly during the first trimester of pregnancy. However, in two observational studies encompassing more than 1800 pregnancies, HU use during conception and pregnancy was not associated with an increased risk of fetal malformations or congenital anomalies, although there was an association with spontaneous abortion and low birth weight [40,44]. The British Haematological Society has recommend the initiation of HU after organogenesis (8 weeks’ gestation) in women with SCD who are at high risk of complications during pregnancy [45], explaining the risks and benefits to the patients. However, no trials have looked at the effectiveness of HU on maternal, fetal, and neonatal outcomes.

No teratogenic effects have been observed in pregnancies where the father was using HU at the time of conception [46]. Although research on HU use in pregnant individuals with SCD is limited, ongoing retrospective studies aim to evaluate the effects of HU during pregnancy and lactation (NCT04093986).

Lactation is currently contraindicated for women with SCD undergoing HU therapy, despite limited pharmacokinetic data. In a study by Ware RE et al., involving 16 lactating volunteers, HU was detected in breast milk with a relative infant dosage of 3.4%, below the recommended safety threshold of 5% to 10% [47,48]. Therefore, breastfeeding may be permitted for women taking daily oral HU.

## 6. Hydroxyurea and Secondary Malignancies

Although the efficacy of HU in managing SCD is well established, uncertainties remain regarding the long-term adverse effects of this treatment.

A systematic review and meta-analysis investigating the use of HU in patients with benign conditions found no cumulative evidence to date that links HU to an increased risk of secondary malignancies or myelodysplastic syndrome [49]. This analysis included 37 studies, including 3 randomized controlled trials (RCTs) and 34 observational studies, with a total of 3278 patients. HU had been used for at least two years in four benign conditions that met the inclusion criteria: sickle cell anemia (SCA) (26 studies), β-thalassemia (9 studies), cyanotic congenital heart diseases (1 study), and renal stones (1 study). In these patient populations, HU was not associated with an increased risk of cancer, with an overall cancer risk (OCR) of 0.2% (95% CI, 0.0–0.3%). Furthermore, 12 studies, or extension studies with follow-up periods of five or more years, demonstrated a stable result of 0.2%. This weighted effect size corresponds to a cancer incidence rate of 2 per 1000 patients, which is consistent with the most recent cancer incidence rates reported for African Americans and Whites in the United States during the 2005–2009 period.

The genotoxic potential of HU has also been evaluated in vitro among patients with SCD who were treated with HU, compared to those who were not treated. Almeida et al. assessed DNA damage in peripheral blood mononuclear cells from 22 SCD patients treated with HU and a control group of 24 SCD patients who had not received HU. PB mononuclear cells were isolated to evaluate genotoxicity using the cytokinesis-block micronucleus cytome assay, in which DNA damage biomarkers—micronuclei, nucleoplasmic bridges, and nuclear buds—were quantified. The mean HU dose in the study was 12.8 mg/kg/day, administered over a mean period of 44 months. The mean micronucleus frequency per 1000 cells was 8.591 ± 1.568 in the HU group and 10.040 ± 1.003 in the control group. Similarly, the mean frequencies of nucleoplasmic bridges and nuclear buds per 1000 cells were 0.4545 ± 0.1707 versus 0.5833 ± 0.2078 and 0.8182 ± 0.2430 versus 0.9583 ± 0.1853 for the HU-treated group and the control group, respectively. There were no statistically significant differences between the two groups [50].

In pediatric patients, Rodriguez et al. investigated the genotoxicity of HU in 76 children: 32 SCD patients treated with HU, 27 SCD patients not treated with HU, and 17 unaffected children [51]. DNA damage was assessed using the comet assay, which enabled the comparison of DNA damage among the three groups. The maximal concentration (Cmax) of HU in plasma was determined following drug administration. The mean values of DNA in the comet tail were 5.13 ± 6.84 for unaffected children, 5.80 ± 7.78 for SCD patients treated with HU, and 5.61 ± 6.91 for SCD patients not treated with HU. Significant differences were observed between unaffected children and SCD patients; however, there were no significant differences in DNA damage between SCD patients treated with HU and those not treated with HU. The authors concluded that SCD patients treated with HU did not exhibit greater nucleoid damage compared to those who were not treated with HU [51].

## 7. Other Sickle Cell Disease Therapies

While HU has been a cornerstone in the treatment of SCD for over two decades, several newer therapies have emerged, offering alternative approaches to managing this complex condition. Novel approaches to treating SCD can target different pathophysiological pathways.

### 7.1. L-Glutamine

L-glutamine has been introduced as an oral supplement. Its mechanism of action involves replenishing cellular antioxidants, thereby enhancing the antioxidant defenses within cells and mitigating the damaging effects of reactive oxygen species. Additionally, L-glutamine is known to enhance the production of nitric oxide, a crucial molecule involved in vasodilation.

Clinical studies have demonstrated positive outcomes with L-glutamine treatment in managing SCD. Evidence suggests that L-glutamine therapy has been shown to significantly lower the incidence of VOCs, ACS, and overall hospital stays. Furthermore, treatment with L-glutamine is associated with improved clinical outcomes, including decreased hemolysis markers, increased hemoglobin levels, and reduced hospital admissions and VOCs [52,53].

In a recent article by Narcisse, Elenga, Gylna, and Loko, real-world data highlighted L-glutamine’s efficacy in alleviating various clinical manifestations of SCD. The study reported a significant reduction in pain crises, hospitalizations, and blood transfusions at 24, 48, and 72 weeks following the initiation of therapy [53].

While L-glutamine treatment is generally well tolerated, it has been associated with side effects, including abdominal pain, nausea, and vomiting, as well as more severe reactions such as splenomegaly, indigestion, and hot flashes [54]. It is noteworthy that L-glutamine has received approval from the FDA but not from the EMA.

### 7.2. Crizanlizumab

Crizanlizumab is a monoclonal antibody that acts as a P-selectin inhibitor, targeting critical mechanisms involved in vaso-occlusive events in SCD. P-selectin, an adhesion molecule expressed on endothelial cells and platelets, plays a pivotal role in the adhesion of red blood cells (RBCs) to the vascular endothelium. This interaction promotes the entrapment and occlusion of RBCs within the microvasculature, contributing to VOCs [55,56]. By binding to P-selectin and blocking its active site, crizanlizumab prevents the adhesion between RBCs, platelets, and endothelial cells, thereby reducing both the frequency and severity of VOCs [55,57]. Additionally, crizanlizumab’s therapeutic benefits extend to reducing inflammation and endothelial activation.

Evidence of crizanlizumab’s efficacy comes from a Phase 2 open-label clinical trial by Kanter et al., which demonstrated a significant reduction in the frequency of VOCs, with a favorable safety profile [56]. Furthermore, a cross-sectional study by Newman et al. underscored the growing use of crizanlizumab in the SCD population, highlighting its potential in reducing pain crises. However, unmet needs within the SCD community remain [57].

The pivotal SUSTAIN trial (NCT01895361) further established crizanlizumab’s effectiveness in reducing VOC frequency in patients with SCD. The drug’s mechanism of action—selective inhibition of P-selectinprevents the adhesion of sickle cells, endothelial cells, and leukocytes, thereby reducing both inflammatory responses and microvascular obstruction [58].

In 2020, the European Medicines Agency (EMA) approved crizanlizumab, marketed as Adakveo, for the prevention of recurrent VOCs in patients aged 16 years and older with SCD. However, in June 2023, the EMA’s Committee for Medicinal Products for Human Use recommended revoking this marketing authorization. This decision followed the evaluation of data from the Phase 3 STAND study (NCT03814746), which compared crizanlizumab with placebo in preventing VOCs in patients with sickle cell anemia. The study did not demonstrate a significant reduction in VOC frequency. Patients treated with crizanlizumab experienced an average of 2.5 painful episodes requiring medical attention during the first year of treatment, compared to 2.3 episodes in the placebo group. Additionally, the average number of painful episodes treated at home was 4.7 for the crizanlizumab group, versus 3.9 in the placebo group.

This recent evidence has raised questions about crizanlizumab’s long-term efficacy, suggesting that further studies are needed to clarify its role in SCD management.

### 7.3. Voxelotor

Voxelotor functions by inhibiting the polymerization of HbS, achieved through its selective binding to HbS and stabilizing it in its oxygenated state. This action reduces the formation of HbS polymers, preventing the sickling of red blood cells. As a result, voxelotor decreases blood viscosity and improves the deformability of red blood cells, leading to enhanced blood flow and a reduction in the incidence of VOCs. Additionally, inhibiting HbS polymerization may reduce hemolysis, oxidative stress, and endothelial dysfunction, contributing to the overall efficacy of voxelotor in managing SCD [59,60].

In a Phase 3 randomized controlled trial, Vichinsky et al. demonstrated that voxelotor significantly increased hemoglobin levels in patients with SCD, while also reducing the risk of hemolysis and anemia exacerbation. The study highlighted the hematological benefits of voxelotor and suggested potential synergistic effects when combined with HU [61]. Shah et al. further emphasized voxelotor’s capacity to decrease transfusion requirements, hospital stays, and hospitalizations related to VOCs in clinical settings [62].

Voxelotor has emerged as a promising therapeutic option for individuals with SCD, supported by clinical studies such as the Phase 3 HOPE trial. Patients treated with voxelotor reported few severe or major side effects after six months of therapy [63]. Common adverse events included gastrointestinal symptoms like diarrhea, nausea, and vomiting, as well as abdominal discomfort, fever, rash, and headache, with over 10% of participants experiencing these effects. Importantly, anaphylaxis occurred in less than 1% of cases, indicating its rarity. No adverse effects specifically associated with cardiovascular or respiratory systems were identified [63,64]. However, it is worth noting that the clinical trials to date have been relatively small and did not include patients with significant comorbidities [65].

Voxelotor received approval from the European Medicines Agency (EMA) in February 2022. However, in a precautionary move, the EMA’s Committee for Medicinal Products for Human Use (CHMP) recently recommended the suspension of voxelotor (Oxbryta)’s marketing authorization. This recommendation followed emerging safety data from two registry-based studies, which indicated an increased frequency of VOC episodes during voxelotor treatment compared to the frequency prior to its initiation. This safety signal emerged during an ongoing review by the EMA, initiated in July 2024, to reassess the benefit–risk profile of Oxbryta. The review was prompted by findings from a clinical trial that reported a higher number of deaths in patients receiving voxelotor compared to those on placebo. Additionally, another trial documented a higher-than-expected mortality rate among participants.

### 7.4. Current Treatment Landscape

Several drugs are currently in advanced stages of development within the therapeutic landscape of sickle cell disease (SCD). Notably, second-generation agents such as inclacumab, a human anti-P-selectin monoclonal antibody, are under investigation; its Phase 3 study has successfully completed patient recruitment (NCT04935879). Additionally, osivelotor is being explored as a potential next-generation successor to voxelotor, with the aim of achieving enhanced therapeutic efficacy at lower dosages (NCT05431088).

Another promising class of compounds comprises pyruvate kinase activators, which stimulate an enzyme critical to cellular metabolism, thereby enhancing cellular energy levels and improving the oxygen-carrying capacity of hemoglobin. These agents exhibit a dual mechanism of action: they increase adenosine triphosphate (ATP) levels in red blood cells, promoting their survival, while simultaneously reducing levels of 2,3-diphosphoglycerate, which enhances hemoglobin’s affinity for oxygen, consequently decreasing polymerization and the formation of sickle cells. Three oral molecules are currently undergoing evaluation: mitapivat, AG-946 (a more potent and selective derivative of mitapivat), and etavopivat. The RISE UP study, a Phase 2/3 trial comparing mitapivat to placebo (NCT05031780), has demonstrated promising results in terms of increasing hemoglobin levels with a favorable safety profile. Recruitment for AG-946 (NCT04536792) has been completed, although the results have yet to be published. Etavopivat is being developed for use in both SCD and thalassemic patients (NCT04624659 and NCT04987489) and is still in the recruitment phase.

Alternative strategies focus on elevating fetal hemoglobin (HbF), akin to hydroxyurea, alongside novel therapeutic agents. AB1, a non-cytotoxic, orally administered anti-disease-modifying therapy (DMT), is currently in Phase 1/2 (NCT05261711) but is on hold pending a protocol amendment to adjust dosing. Complement inhibitors may also play a significant role in the pathophysiology of vaso-occlusion; crovalimab, a C5 inhibitor, is under development, with several clinical trials currently in progress (NCT04912869, NCT05075824).

Experts advocate for future trials exploring combination therapies tailored to specific complications such as vaso-occlusive crises (VOCs), chronic pain, and renal impairment, as well as a personalized medicine approach grounded in patient phenotypes and characteristics. Following the recent approval of gene therapy for SCD, a pivotal challenge lies in addressing the importance of shared decision-making with families, alongside considerations of global access and affordability.

### 7.5. Allogeneic Stem-Cell Transplantation

Allogeneic hematopoietic cell transplantation (HCT) has been recognized as a curative therapy for SCD. Long-term studies have demonstrated a disease-free survival rate of over 90%, particularly among younger patients with well-matched donors. This makes allogeneic HCT a highly effective treatment option for carefully selected patients.

A landmark review by Walters et al. in 2016 extensively examined the indications and outcomes of HCT from HLA-identical sibling donors for SCD. The review consolidated data from multiple studies, revealing an overall survival rate of 95% and an event-free survival rate of 92% among children who underwent HCT with HLA-identical sibling donors. The authors emphasized the importance of early intervention and careful patient selection, considering factors such as disease severity and the availability of an optimal donor [66].

A recent study published in 2023 further explored long-term outcomes following unrelated donor HCT for severe SCD, as part of the Bone Marrow Transplant Clinical Trials Network (BMT CTN) 0601 trial. This study included 21 pediatric patients and reported that 68% were alive approximately eight years post-transplant, with 57% reporting good overall health. However, severe complications, such as graft-versus-host disease (GVHD), were observed, particularly among patients older than 13 years at the time of transplantation. The study underscored the importance of matched sibling donors for optimal outcomes, while also suggesting that matched unrelated donors could be a feasible alternative when sibling donors are not available [67].

Despite the promising outcomes associated with allogeneic HCT, complications such as GVHD and treatment-related mortality remain significant concerns. The success of allo-HCT is closely tied to donor compatibility and the patient’s health status at the time of transplantation. As advancements in gene therapy continue to evolve, allogeneic HCT remains a critical therapeutic option in the management of severe SCD, particularly in cases where suitable candidates for gene therapy are not available.

### 7.6. Gene Therapy

Gene therapy offers a promising yet experimental approach to the treatment of SCD. Advanced techniques such as CRISPR-Cas9 and lentiviral gene editing aim to correct the underlying genetic mutation responsible for SCD or induce the production of HbF by disrupting the BCL11A gene [68,69]. Early clinical trials have shown remarkable outcomes, with many patients experiencing a near-complete cessation of VOCs and achieving transfusion independence.

Currently, two types of gene therapy are under investigation for the treatment of SCD [70]:

○ Exagamglogene Autotemcel (Exa-cel): This approach focuses on gene addition to correct abnormal hemoglobin production, thereby improving cellular function. This method was evaluated in clinical trial NCT03745287, which included 47 patients. The trial has been completed, demonstrating a significant reduction in VOCs (*p* < 0.001) and improvements in quality of life.
○ Lovotibeglogene Autotemcel (Lovo-cel): This therapy utilizes an HbF induction strategy, promoting a shift in red blood cell production and alleviating disease symptoms. Clinical trials NCT02140554 (HGB-206) and NCT04293185 (HGB-210) studied a total of 46 patients. Both trials have been completed, with 91% of patients achieving transfusion independence and experiencing a significant reduction in VOCs (*p* < 0.05).

However, these clinical trials did not perform a direct comparison with HU, a standard treatment for SCD, making it challenging to establish a definitive advantage of gene therapy over HU. Additionally, gene therapy is limited by high costs, technical complexity, and the need for myeloablative conditioning, which carries considerable risks. In contrast, HU remains an affordable, widely accessible treatment with a well-established safety profile, making it a more practical option for the majority of SCD patients, particularly in low-resource settings.

## 8. Conclusions

Hydroxyurea is the cornerstone of therapeutic management for sickle cell disease. It significantly reduces vaso-occlusive crises, acute chest syndrome, and the need for transfusions. This leads to lower morbidity and mortality rates. Despite its well-established benefits, concerns about long-term adverse effects persist, especially regarding fertility and potential genotoxicity. Current evidence indicates that HU does not increase the risk of secondary malignancies or myelodysplastic syndrome, and it does not cause DNA damage in treated patients. However, further studies are needed to fully understand its safety, particularly during pregnancy and lactation, where the risks remain unclear. Given its significant clinical advantages, including long-term efficacy, a favorable safety profile, cost-effectiveness, and disease-modifying potential, the early and continuous use of HU is strongly recommended in the management of SCD.

Nonetheless, therapeutic decisions should consider individual patient circumstances, such as pregnancy.

Given the recent withdrawal of alternative pharmacological treatments such as crizanlizumab and voxelotor from the market by the European Medicines Agency (EMA), the primary alternatives to HU therapy now include allogeneic hematopoietic stem-cell transplantation and emerging gene therapy approaches.

## Figures and Tables

**Table 1:** Hydroxyurea treatment initiation protocol [20].

Phase	Action/Recommendation
Pre-therapy laboratory tests	- Complete blood count (CBC) with differential (WBC, reticulocyte count, platelet count, RBC mean corpuscular volume). - Fetal hemoglobin measurement (if possible). - Renal and liver function tests. - Pregnancy test (for women).
Therapy initiation	- Baseline elevated fetal hemoglobin levels should not delay therapy initiation. - Counsel reproductive-age patients on contraception needs during therapy. - Start adults at 15 mg/kg/d (500 mg capsules); 5–10 mg/kg/d if chronic kidney disease is present. - Start infants/children at 20 mg/kg/d.
Monitoring and adjustments	- CBC with differential and reticulocyte count every 4 weeks when adjusting dosage. - Aim for absolute neutrophil count ≥ 2000/µL; younger patients may tolerate counts down to 1250/µL. - Maintain platelet count ≥ 80,000/µL.
Response to cytopenia	- If neutropenia/thrombocytopenia occurs: ○ Stop HU temporarily; ○ Monitor CBC weekly; ○ Reintroduce HU at 5 mg/kg/d lower dose once counts normalize.
Dose escalation	- If warranted, increase dose by 5 mg/kg/d every 8 weeks until mild myelosuppression (neutrophil count of 2000–4000/µL) is achieved, up to 35 mg/kg/d.
Long-term monitoring	- Regular safety checks every 2–3 months once stable dosing is achieved: CBC, reticulocyte and platelet counts. - Monitor RBC MCV and fetal hemoglobin levels for consistent response.
Additional considerations	- Clinical response may take 3–6 months; continue on max dose for 6 months before evaluating discontinuation. - Long-term therapy recommended even during hospitalizations or illness.

**Table 2:** Treatment adjustment criteria according to toxicities [4].

**Treatment discontinuation criteria**
Severe gastrointestinal side effects
Severe skin rash
Malleolar ulcers
Pregnancy and breastfeeding
**Factors requiring dose reduction**
Nausea
Vomiting
Mucositis
Diarrhea
Alopecia
Skin rash
**Temporary discontinuation criteria**
Acute complications with cytopenias or severe renal function impairment

## References

[1] Sundd P., Gladwin M.T., Novelli E.M. (2019). Pathophysiology of Sickle Cell Disease. Annu. Rev. Pathol. Mech. Dis..

[2] Argüello Marina M., Villegas Martínez A. (2021). Fisiopatología de la REDopatía S. Guía de Enfermedad de Células Falciformes.

[3] Thomson A.M., McHugh T.A., Oron A.P., Teply C., Lonberg N., Tella V.V., Wilner L.B., Fuller K., Hagins H., Aboagye R.G. (2023). Global, regional, and national prevalence and mortality burden of sickle cell disease, 2000–2021: A systematic analysis from the Global Burden of Disease Study 2021. Lancet Haematol..

[4] López Rubio M., Morado Arias M., Ricard Andrés M.P., Villegas Martínez A. (2021). Guía de Enfermedad de Células Falciformes.

[5] Amuel S., Harache C., Errin I.L.T., Oore I.D.M., Eorge G., Over J.D., Barton F.B., Eckert S.V., McMahon R.P., Bonds D.R. (1995). Effect of hydroxyurea on the frequency of painful crises in sickle cell anemia and the investigators of the multicenter study of hydroxyurea in sickle cell anemia. N. Engl. J. Med..

[6] Voskaridou E., Christoulas D., Bilalis A., Plata E., Varvagiannis K., Stamatopoulos G., Sinopoulou K., Balassopoulou A., Loukopoulos D., Terpos E. (2010). The effect of prolonged administration of hydroxyurea on morbidity and mortality in adult patients with sickle cell syndromes: Results of a 17-year, single-center trial (LaSHS). Blood.

[7] Steinberg M.H., Barton F., Castro O., Pegelow C.H., Ballas S.K., Kutlar A., Orringer E., Bellevue R., Olivieri N., Eckman J. Effect of Hydroxyurea on Mortality and Morbidity in Adult Sickle Cell Anemia Risks and Benefits Up to 9 Years of Treatment [Internet]. http://jama.jamanetwork.com/.

[8] Surendra Baliga B., Pace B.S., Chen H.H., Shah A.K., Yang Y.M. (2000). Mechanism for fetal hemoglobin induction by hydroxyurea in sickle cell erythroid progenitors. Am. J. Hematol..

[9] McGann P.T., E Ware R. (2015). Hydroxyurea therapy for sickle cell anemia. Expert Opin. Drug Saf..

[10] Brandow A.M., Liem R.I. (2022). Advances in the diagnosis and treatment of sickle cell disease. J. Hematol. Oncol..

[11] Strouse J.J., Lanzkron S., Beach M.C., Haywood C., Park H., Witkop C., Wilson R.F., Bass E.B., Segal J.B. (2008). Hydroxyurea for Sickle Cell Disease: A Systematic Review for Efficacy and Toxicity in Children. Pediatrics.

[12] Cokic V.P., Smith R.D., Beleslin-Cokic B.B., Njoroge J.M., Miller J.L., Gladwin M.T., Schechter A.N. (2003). Hydroxyurea induces fetal hemoglobin by the nitric oxide–dependent activation of soluble guanylyl cyclase. J. Clin. Investig..

[13] Gambero S., Canalli A.A., Traina F., Albuquerque D.M., Saad S.T., Costa F.F., Conran N. (2007). Therapy with hydroxyurea is associated with reduced adhesion molecule gene and protein expression in sickle red cells with a concomitant reduction in adhesive properties. Eur. J. Haematol..

[14] Almeida C.B., Scheiermann C., Jang J.E., Prophete C., Costa F.F., Conran N., Frenette P.S. (2012). Hydroxyurea and a cGMP-amplifying agent have immediate benefits on acute vaso-occlusive events in sickle cell disease mice. Blood J. Am. Soc. Hematol..

[15] Ferster A., Vermylen C., Cornu G., Buyse M., Corazza F., Devalck C., Fondu P., Toppet M., Sariban E. (1996). Hydroxyurea for treatment of severe sickle cell anemia: A pediatric clinical trial. Blood.

[16] Thornburg C.D., Files B.A., Luo Z., Miller S.T., Kalpatthi R., Iyer R., Seaman P., Lebensburger J., Alvarez O., Thompson B. (2012). Impact of hydroxyurea on clinical events in the BABY HUG trial. Blood.

[17] Wang W.C., E Ware R., Miller S.T., Iyer R.V., Casella J.F., Minniti C.P., Rana S., Thornburg C.D., Rogers Z.R., Kalpatthi R.V. (2011). Hydroxycarbamide in very young children with sickle-cell anaemia: A multicentre, randomised, controlled trial (BABY HUG). Lancet.

[18] Jain D.L., Sarathi V., Desai S., Bhatnagar M., Lodha A. (2012). Low Fixed-Dose Hydroxyurea in Severely Affected Indian Children with Sickle Cell Disease. Hemoglobin.

[19] Khargekar N., Banerjee A., Athalye S., Mahajan N., Kargutkar N., Tapase P., Madkaikar M. (2024). Role of hydroxyurea therapy in the prevention of organ damage in sickle cell disease: A systematic review and meta-analysis. Syst. Rev..

[20] Yawn B.P., Buchanan G.R., Afenyi-Annan A.N., Ballas S.K., Hassell K.L., James A.H., Jordan L., Lanzkron S., Lottenberg R., Savage W.J. (2014). Management of Sickle Cell Disease: Summary of the 2014 Evidence-Based Report by Expert Panel Members. JAMA.

[21] Hankins J.S., Helton K.J., McCarville M.B., Li C., Wang W.C., Ware R.E. (2008). Preservation of spleen and brain function in children with sickle cell anemia treated with hydroxyurea. Pediatr. Blood Cancer.

[22] Wang W.C., Wynn L.W., Rogers Z.R., Scott J., Lane P.A., Ware R.E. (2001). A two-year pilot trial of hydroxyurea in very young children with sickle-cell anemia. J. Pediatr..

[23] Schuchard S.B., Lissick J.R., Nickel A., Watson D., Moquist K.L., Blaylark R.M., Nelson S.C. (2019). Hydroxyurea use in young infants with sickle cell disease. Pediatr. Blood Cancer.

[24] Ware R.E., McGann P.T., Quinn C.T. (2019). Hydroxyurea for children with sickle cell anemia: Prescribe it early and often. Pediatr. Blood Cancer.

[25] Ware R.E., Davis B.R., Schultz W.H., Brown R.C., Aygun B., Sarnaik S., Odame I., Fuh B., George A., Owen W. (2016). Hydroxycarbamide versus chronic transfusion for maintenance of transcranial doppler flow velocities in children with sickle cell anaemia—TCD with Transfusions Changing to Hydroxyurea (TWiTCH): A multicentre, open-label, phase 3, non-inferiority trial. Lancet.

[26] Casella J.F., Furstenau D.K., Adams R.J., Brambilla D.J., Lebensburger J.D., Fehr J.J., Jordan L.C., King A.A., Ichord R.N., McKinstry R.C. (2024). Hydroxyurea to prevent brain injury in children with sickle cell disease (HU Prevent)—A randomized, placebo-controlled phase II feasibility/pilot study. Am. J. Hematol..

[27] Lai J., Zou P., da Rocha J.L.D., Heitzer A.M., Patni T., Li Y., Scoggins M.A., Sharma A., Wang W.C., Helton K.J. (2024). Hydroxyurea maintains working memory function in pediatric sickle cell disease. PLoS ONE.

[28] Luchtman-Jones L., Pressel S., Hilliard L., Brown R.C., Smith M.G., Thompson A.A., Lee M.T., Rothman J., Rogers Z.R., Owen W. (2016). Effects of hydroxyurea treatment for patients with hemoglobin SC disease. Am. J. Hematol..

[29] Alkindi S., Al-Busaidi I.B.M., Pathare A.V. (2024). Clinical and Laboratory Features of Sickle Cell Disease S/D Punjab: Impact of HbF and Hydroxyurea. Mediterr J. Hematol. Infect. Dis..

[30] Ware R.E. (2010). How I use hydroxyurea to treat young patients with sickle cell anemia. Blood.

[31] Ogu U.O., Mukhopadhyay A., Patel K., Nelson M.N., Strahan K.S., Wu L., Smeltzer M.P., Ataga K.I. (2024). Hydroxyurea at escalated dose versus fixed low-dose hydroxyurea in adults with sickle cell disease. Eur. J. Haematol..

[32] Wang W.C., Brown R.C., McNaull M.A., Rogers Z.R., Barton M., Dua M.R., Hankins J.S., Gossett J., Richardson J., Porter J.S. (2023). Intensive hydroxyurea dosing in very young children with sickle cell anemia. Blood Adv..

[33] Houwing M.E., Bos J., van Wijk R., van Beers E.J., Cnossen M.H., Rab M.A.E., the SCORE consortium (2024). Use of the oxygen gradient ektacytometry in the dose titration of hydroxyurea therapy in children with sickle cell disease. Int. J. Lab. Hematol..

[34] Smaldone A., Manwani D., Aygun B., Appiah-Kubi A., Smith-Whitley K., Green N.S. (2024). Assessing multilevel barriers to hydroxyurea adherence in youth with sickle cell disease using pharmacy-based refill records. Pediatr. Blood Cancer.

[35] Walden J., Brown L., Seiguer S., Munshaw K., Rausch J., Badawy S., McGann P., Winkler S., Gonzalez L., Creary S. (2024). Study protocol for ADHERE (Applying Directly observed therapy to HydroxyurEa to Realize Effectiveness): Using small business partnerships to deliver a scalable and novel hydroxyurea adherence solution to youth with sickle cell disease. PLoS ONE.

[36] Roessner C., Sale T., Uminski K., Goodyear D., Rydz N. (2024). A pharmacist-managed hydroxyurea prescribing protocol improves uptake and optimization among patients with sickle cell disease. Adv. Hematol..

[37] Zimmerman S.A., Schultz W.H., Davis J.S., Pickens C.V., Mortier N.A., Howard T.A., Ware R.E. (2004). Sustained long-term hematologic efficacy of hydroxyurea at maximum tolerated dose in children with sickle cell disease. Blood.

[38] Estepp J.H., Smeltzer M.M.P., Kang G., Aygun B., Ware R.E., Nottage K. (2014). Higher Fetal Hemoglobin Following Escalation of Hydroxyurea to Maximum Tolerated Dose Provides Clinical Benefit to Children with Sickle Cell Anemia. Blood.

[39] DeBaun M.R. (2014). Hydroxyurea therapy contributes to infertility in adult men with sickle cell disease: A review. Expert Rev. Hematol..

[40] Kroner B.L., Hankins J.S., Pugh N., Kutlar A., King A.A., Shah N.R., Kanter J., Glassberg J., Treadwell M., Gordeuk V.R. (2022). Pregnancy outcomes with hydroxyurea use in women with sickle cell disease. Am. J. Hematol..

[41] Lukusa A., Vermylen C. (2008). Use of hydroxyurea from childhood to adult age in sickle cell disease: Semen analysis. Haematologica.

[42] Sewaralthahab S., Alsubki L.A., Alhrabi M.S., Alsultan A. (2024). Effects of hydroxyurea on fertility in male and female sickle cell disease patients: A systematic review and meta-analysis. PLoS ONE.

[43] Diesch-Furlanetto T., Poirot C. Impact of Hydroxyurea on Follicle Density in Patients with Sickle Cell Disease. https://pubmed.ncbi.nlm.nih.gov/39023361/.

[44] Habibi A., Cannas G., Bartolucci P., Voskaridou E., Joseph L., Bernit E., Gellen-Dautremer J., Charneau C., Ngo S., Galactéros F. (2023). Outcomes of Pregnancy in Sickle Cell Disease Patients: Results from the Prospective ESCORT-HU Cohort Study. Biomedicines.

[45] Sinkey R.G., Ogunsile F.J., Kanter J., Bean C., Greenberg M. (2024). Society for Maternal-Fetal Medicine Consult Series #68: Sickle cell disease in pregnancy. Am. J. Obstet. Gynecol..

[46] Ballas S.K., McCarthy W.F., Guo N., DeCastro L., Bellevue R., Barton B.A., Waclawiw M.A., The Investigators of the Multicenter Study of Hydroxyurea in Sickle Cell Anemia (2009). Exposure to hydroxyurea and pregnancy outcomes in patients with sickle cell anemia. J. Natl. Med. Assoc..

[47] Ware R.E., Marahatta A., Ware J.L., McElhinney K., Dong M., Vinks A.A. (2020). Hydroxyurea Exposure in Lactation: A Pharmacokinetics Study (HELPS). J. Pediatr..

[48] Dong M., Ware R.E., Dallmann A., Vinks A.A. (2023). Hydroxyurea treatment for sickle cell anemia during pregnancy and lactation: Current evidence and knowledge gaps. Pharmacotherapy.

[49] Algiraigri A.H., Radwi M. (2014). Long-Term Safety of Hydroxyurea in Sickle Cell Anemia and Other Benign Diseases: Systematic Review and Meta-Analysis. Blood.

[50] Oliveira EA M.D., Boy KD A., Santos AP P., Machado CD S., Velloso-Rodrigues C., Gerheim PS A.S., Mendonça L.M. (2019). Evaluation of hydroxyurea genotoxicity in patients with sickle cell disease. Einstein.

[51] Rodriguez A., Duez P., Dedeken L., Cotton F., Ferster A. (2018). Hydroxyurea (hydroxycarbamide) genotoxicity in pediatric patients with sickle cell disease. Pediatr. Blood Cancer.

[52] Goubran Messiha H., Alali A., Elalfy M.S., Yassin M.A. Real-World Data on Efficacy of L-Glutamine in Preventing Sickle Cell Disease-Related Complications in Pediatric and Adult Patients. https://www.frontiersin.org/articles/10.3389/fmed.2022.931925/full.

[53] Migotsky M., Beestrum M., Badawy S.M. (2022). Recent Advances in Sickle-Cell Disease Therapies: A Review of Voxelotor, Crizanlizumab, and L-glutamine. Pharmacy.

[54] Sadaf A., Quinn C.T. (2020). L-glutamine for sickle cell disease: Knight or pawn?. Exp. Biol. Med..

[55] Man Y., Goreke U., Kucukal E., Hill A., An R., Liu S., Bode A., Solis-Fuentes A., Nayak L.V., Little J.A. (2020). Leukocyte adhesion to P-selectin and the inhibitory role of Crizanlizumab in sickle cell disease: A standardized microfluidic assessment. Blood Cells, Mol. Dis..

[56] Kanter J., Brown R.C., Norris C., Nair S.M., Kutlar A., Manwani D., Shah N., Tanaka C., Bodla S., Sanchez-Olle G. (2023). Pharmacokinetics, pharmacodynamics, safety, and efficacy of crizanlizumab in patients with sickle cell disease. Blood Adv..

[57] Newman T.V., Yang J., Suh K., Jonassaint C.R., Kane-Gill S.L., Novelli E.M. (2023). Use of disease-modifying treatments in patients with sickle cell disease. JAMA Netw. Open.

[58] Ataga K.I., Kutlar A., Kanter J., Liles D., Cancado R., Friedrisch J., Guthrie T.H., Knight-Madden J., Alvarez O.A., Gordeuk V.R. (2017). Crizanlizumab for the Prevention of Pain Crises in Sickle Cell Disease. N. Engl. J. Med..

[59] Howard J., Hemmaway C.J., Telfer P., Layton D.M., Porter J., Awogbade M., Mant T., Gretler D.D., Dufu K., Hutchaleelaha A. (2019). A Phase 1/2 Ascending Dose Study and Open-Label Extension Study of Voxelotor in Patients with Sickle Cell Disease [Internet]. www.clinicaltrials.gov.

[60] Phan V., Hershenson J., Caldarera L., Larkin S.K., Wheeler K., Cortez A.L., Dulman R., Briere N., Lewis A., Kuypers F.A. (2023). Effect of voxelotor on cardiopulmonary testing in youths with sickle cell anemia in a pilot study. Pediatr. Blood Cancer.

[61] Vichinsky E., Hoppe C.C., Ataga K.I., Ware R.E., Nduba V., El-Beshlawy A., Hassab H., Achebe M.M., Alkindi S., Brown R.C. (2019). A Phase 3 Randomized Trial of Voxelotor in Sickle Cell Disease. N. Engl. J. Med..

[62] Estepp J.H., Kalpatthi R., Woods G., Trompeter S., Liem R.I., Sims K., Inati A., Inusa B.P.D., Campbell A., Piccone C. (2022). Safety and efficacy of voxelotor in pediatric patients with sickle cell disease aged 4 to 11 years. Pediatr. Blood Cancer.

[63] Dufu K., Patel M., Oksenberg D., Cabrales P. (2018). GBT440 improves red blood cell deformability and reduces viscosity of sickle cell blood under deoxygenated conditions. Clin. Hemorheol. Microcirc..

[64] Telfer P., Agodoa I., Fox K.M., Burke L., Mant T., Jurek M., Tonda M., Lehrer-Graiwer J. (2018). Impact of voxelotor (GBT440) on unconjugated bilirubin and jaundice in sickle cell disease: A patient case report. Hematol. Rep..

[65] Tayyaba Rehan S., Hussain H ul Malik F., Usama R.M., Tahir M.J., Asghar M.S. (2022). Voxelotor versus other therapeutic options for sickle cell disease: Are we still lagging behind in treating the disease?. Health Sci. Rep..

[66] Walters M.C., De Castro L.M., Sullivan K.M., Krishnamurti L., Kamani N., Bredeson C., Neuberg D., Hassell K.L., Farnia S., Campbell A. (2016). Indications and Results of HLA-Identical Sibling Hematopoietic Cell Transplantation for Sickle Cell Disease. Biol. Blood Marrow Transplant..

[67] Eapen M., Kou J., Andreansky M., Bhatia M., Brochstein J., Chaudhury S., Haight A.E., Haines H., Jacobsohn D., Jaroscak J. (2024). Long-term outcomes after unrelated donor transplantation for severe sickle cell disease on the BMT CTN 0601 trial. Am. J. Hematol..

[68] Esrick E.B., Lehmann L.E., Biffi A., Achebe M., Brendel C., Ciuculescu M.F., Daley H., MacKinnon B., Morris E., Federico A. (2021). Post-Transcriptional Genetic Silencing of *BCL11A* to Treat Sickle Cell Disease. N. Engl. J. Med..

[69] Frangoul H., Altshuler D., Cappellini M.D., Chen Y.S., Domm J., Eustace B.K., Foell J., de la Fuente J., Grupp S., Handgretinger R. (2021). CRISPR-Cas9 Gene Editing for Sickle Cell Disease and β-Thalassemia. N. Engl. J. Med..

[70] Sharma A., Jude S. (2023). How I Treat Sickle Cell Disease with Gene Therapy. https://ashpublications.org/hematology/article/2023/1/542/506475/Gene-therapy-for-sickle-cell-disease.

